# Evaluation of Fungal Laccase Immobilized on Natural Nanostructured Bacterial Cellulose

**DOI:** 10.3389/fmicb.2015.01245

**Published:** 2015-11-10

**Authors:** Lin Chen, Min Zou, Feng F. Hong

**Affiliations:** ^1^Group of Microbiological Engineering and Industrial Biotechnology, College of Chemistry, Chemical Engineering, and Biotechnology, Donghua UniversityShanghai, China; ^2^Key Laboratory of High Performance Fibers and Products, Ministry of Education, Donghua UniversityShanghai, China

**Keywords:** bacterial cellulose, laccase, adsorption, cross-linking, immobilization

## Abstract

The aim of this work was to assess the possibility of using native bacterial nanocellulose (BC) as a carrier for laccase immobilization. BC was synthesized by *Gluconacetobacter xylinus*, which was statically cultivated in a mannitol-based medium and was freeze-dried to form BC sponge after purification. For the first time, fungal laccase from *Trametes versicolor* was immobilized on the native nanofibril network-structured BC sponge through physical adsorption and cross-linking with glutaraldehyde. The properties including morphologic and structural features of the BC as well as the immobilized enzyme were thoroughly investigated. It was found that enzyme immobilized by cross-linking exhibited broader pH operation range of high catalytic activity as well as higher running stability compared to free and adsorbed enzyme. Using ABTS as substrate, the optimum pH value was 3.5 for the adsorption-immobilized laccase and 4.0 for the crosslinking-immobilized laccase. The immobilized enzyme retained 69% of the original activity after being recycled seven times. Novel applications of the BC-immobilized enzyme tentatively include active packaging, construction of biosensors, and establishment of bioreactors.

## Introduction

Laccase (benzenediol:oxygen oxidoreductase, EC 1.10.3.2) is a multi-copper oxidase that is widely distributed in plants and certain fungi (Reinhammar, 1997). Laccase catalyzes the one-electron oxidation of a variety of aromatic compounds, in particular phenols, as well as diamines and hexacyanoferrate, concomitantly with the four-electron reduction of molecular oxygen to water. However, in the presence of low-molecular mass mediators, laccase can be employed for the oxidation of a variety of non-phenolic aromatic compounds (Hong et al., 2006; Riva, 2006; Cañas and Camarero, 2010). Thus, laccases have very broad substrate specificity with regard to the reducing substrate and have an interesting potential as industrial enzymes. Laccases are attracting considerable interest for a variety of biotechnological applications, such as in organic synthesis/transformation, textile processing, food industry, pharmaceutical industry, remediation of contaminated environments, delignification of pulp, modification of lignocellulosic materials, manufacture of new materials, as well as construction of biosensors and biofuel cells (Couto and Toca-Herrera, 2006; Munari et al., 2007; Rita et al., 2008; Litescu et al., 2010; Majeau et al., 2010; Kudanga et al., 2011).

Like many other enzymes, laccases are usually limited in practical applications due to their short lifetimes and due to that they are difficult to recover from the reaction system, which might cause high processing cost. To resolve this, enzyme immobilization on insoluble supports is often proposed owing to many advantages such as ready reutilization of the catalyst and possibility of continuous operation. Laccase has been reported to be immobilized on many kinds of carriers, e.g., kaolinite (Hu et al., 2007), ceramic honeycomb (Plagemann et al., 2011), mesostructured silica materials including magnetic mesoporous spheres, or nanoparticles (Zhu et al., 2007; Bautista et al., 2010; Wang et al., 2010) and cellular foams (Rekuć et al., 2009; Zhao et al., 2011), polymer beads (Arica et al., 2009; Makas et al., 2010), agroindustrial residues including coconut fiber (Cristovao et al., 2011) and beer spent grain (da Silva et al., 2012), ionic liquid-modified cellulose acetate (Moccelini et al., 2011), membranes based on epoxy resin (Chawla et al., 2011), chemically modified polypropylene (Georgieva et al., 2010), copper ion-chelated chitosan (Bayramoglu et al., 2012), and plasma-treated cellulosic or polyamide materials (Labus et al., 2012). Immobilization based on these methods either utilize organically synthesized materials, where some organic solvents may be not environmentally friendly, or require laborious operation procedures which give low efficiency in enzyme immobilization.

Bacterial cellulose (BC) is a kind of natural cellulose synthe sized by some bacteria, especially *Gluconacetobacter xylinus* (formerly *Acetobacter xylinus*) (Bielecki et al., 2002). Although chemically identical to plant cellulose, BC is characterized by a unique fibrillar nanostructure which determines its distinguished physical and mechanical properties such as high porosity, large surface area, excellent mechanical strength and good biocompatibility (Bielecki et al., 2002; Gama et al., 2012). Therefore, BC has been used widely for making high-quality audio membranes, electronic paper, membranes for fuel cells, and biomedical materials (Bielecki et al., 2002; Shah and Brown, 2005; Klemm et al., 2006; Petersen and Gatenholm, 2011; Gama et al., 2012; Jiang et al., 2012, 2015; Hong et al., 2015; Tang et al., 2015). Already today, several attempts including development of cost-effective feedstocks from agroindustrial residues (Hong et al., 2011, 2012; Cavka et al., 2013; Chen et al., 2013; Guo et al., 2013, 2015) and establishment of new cultivation methods (Sani and Dahman, 2010) have been made to decrease the production cost of BC. More applications for BC are likely to emerge if the material would become less expensive. To the best of our knowledge, there are no previous studies on immobilization of laccase on nanostructured BC.

For enzyme immobilization, the nanostructured BC material is expected to easily entrap enzyme molecules and achieve high protein loadings because of its large surface area and high porosity. Recent research indicates that untreated or unmodified cellulose-based materials are not capable of immobilizing enzyme. For example, untreated and plasma-treated cellulosic membranes were unsuitable for laccase and tyrosinase immobilization (Labus et al., 2012), and only chemically modified BC pellets (not pristine BC) have successfully been used for glucoamylase immobilization by covalent attachment (Wu and Lia, 2008). Laccase is an enzyme with great biotechno logicalpotential. Therefore the investigation on immobilization of the enzyme using natural BC as a support is highly meaningful because of its novelty, operational convenience, and environmental advantages.

In this work, a BC hydrogel membrane was prepared by *G. xylinus* grown in a mannitol-based medium, and was then freeze-dried to a sponge to maintain its native and unique fibrillar nanostructured network. The spongy membrane, which was prepared without any pretreatment or chemical derivatization, was utilized as the support to immobilize a fungal laccase from *Trametes versicolor* by physical adsorption and by cross-linking. Although the properties of the BC polymer are well-known and the methodologies for enzyme immobilization using adsorption and glutaraldehyde-crosslinking are mature, this is the first time that an enzyme is immobilized on natural nanostructured BC, in a way so that the unique ultrafine fibrillar networks of BC are fully utilized. The properties of the BC and the factors influencing enzyme immobilization were thoroughly investigated. A range of characteristics including optimum catalytic conditions and reuse stability of the immobilized laccases were evaluated and compared with those of free enzyme. The proposed method has several potential benefits including utilization of an environmentally benign and sustainable material, convenience of immobilization using a simple method, improvement of enzyme performance, as well as excellent wet mechanical strength suitable for long-term running in film bioreactors.

## Materials and Methods

### Materials

Laccase from *T. versicolor* and 2,2′-azino-bis-(3- ethylbenz thiazoline-6-sulfonic acid) (ABTS) was obtained from Sigma–Aldrich (Steinheim, Germany). The other chemicals were of analytical grade and commercially available. Determination of laccase activity was performed spectrophotometrically at 414 nm according to a previous study (Hong et al., 2002). One unit of laccase activity corresponds to the amount of enzyme which forms 1 μmol radical cation of ABTS per minute at pH 5.2 and 30°C.

### Preparation of BC Membrane by Bacterial Cultivation

An acetic acid bacterium, *G. xylinus* (formerly *Acetobacter xylinus*) ATCC 23770 was obtained from American Type Culture Collection (Manassas, VA, USA) and used in this study to prepare BC membranes. The culture medium adopted here was mannitol medium, which contained 25 g/L D-mannitol, 5 g/L yeast extract and 3 g/L tryptone with the pH value of 5.0, and was sterilized by autoclaving at 121°C (Hong et al., 2011). The bacteria were grown in a 500 mL flask containing 100 mL of the mannitol medium and cultivated at 30°C statically for 7 days. After cultivation, BC membranes were obtained.

### Purification of Bacterial Cellulose Membrane

After cultivation, the BC membrane was collected, washed with deionized water and incubated in 0.1% NaOH at 80°C for 2 h in order to remove the bacterial cells and residual culture medium embedded in the BC. Thereafter BC membrane was washed in deionized water and incubated again in fresh 0.1% NaOH at 80°C for 2 h. Afterward BC was washed by deionized water until the pH value of the washing liquid became neutral. Finally, the wet membrane was freeze-dried for 24 h and cut into small pieces with a size of 7 mm × 7 mm to 20 mm × 20 mm. The resulting samples were used for enzyme immobilization.

### Immobilization of Laccase

In general, laccase immobilization by adsorption was carried out (Hong et al., 2010) as follows: freeze-dried BC samples was placed into 10 mL of laccase aqueous solution (1000 U/L, pH 5.0) and incubated at 23°C and 80 rpm for 5 min, then was placed in a 4°C fridge statically for 24 h to equilibrate. Afterward the BC gels were filtered out. The surface of the gels were rinsed 1–2 times quickly with sodium acetate buffer (pH 5.2, 200 mM) and dried by tissue paper.

The enzyme immobilized by cross-linking was prepared with the same mixture in the adsorption method, and the mixture was incubated at 4°C and 80 rpm for 24 h. After that, 0.5 mL of 2.5% glutaraldehyde solution was added in the mixture and treated under at 30°C and 100 rpm for 2 h. Finally, the gels were filtered out, and treated using the same procedure as in the adsorption method.

### Assay of Immobilized Laccase Activity

The laccase activity was measured using ABTS (ɛ_ABTS_ = 36,000 M^-1^.cm^-1^) as substrate (Hong et al., 2002). One activity unit was defined as the amount of enzyme that oxidized 1 μmol of ABTS per min. In a typical experiment, 20 mL of reaction medium composed of 0.4 mM ABTS and 50 mM sodium acetate buffer (pH 5.2) was placed into a 100 mL screw-capped vial, which was incubated in a 30°C water bath with 220 rpm agitation on a magnetic stirrer. The reaction was started by adding free or immobilized laccase. Aliquots were withdrawn from the reaction mixture at different reacting intervals and analyzed spectrophotometrically at 414 nm. For the immobilized enzyme, sampling started after 10 min. The activity of immobilized laccase was calculated using the following equation (1). Triplicate experiments for each sample were carried out and mean values are given. Analysis of variance (ANOVA) was performed and *P* value is given.

(1) $$[\mathrm{Activity}\mathrm{of}\mathrm{immobilized}\mathrm{laccase}(U/g\mathrm{BC})=\frac{k\times V_{Total}\times 10^{6}}{M_{BC}\times \varepsilon }],$$

where *k* is the slope of the plot of the absorbance changes at 414 nm versus reaction time,

*V*_Total_ is the total volume of reaction medium (0.02 L),

*M*_BC_ is the mass of BC support of immobilized laccase (g),

ɛ is the extinction coefficient of ABTS (ɛ_ABTS_ = 36,000 M^-1^.cm^-1^).

(2) $$\begin{array}{c} {}[\mathrm{Adsorption}\mathrm{ratio}\mathrm{of}\mathrm{laccase}(\%)=[\mathrm{Total}\mathrm{activity}\mathrm{of}\mathrm{free}\mathrm{laccase}(U)-Total\mathrm{residual}\mathrm{activity}\\ \mathrm{of}\mathrm{free}\mathrm{laccase}\mathrm{after}\mathrm{immobilization}(U)]÷\mathrm{Total}\mathrm{activity}\mathrm{of}\mathrm{free}\mathrm{laccase}(U)\times 100] \end{array}$$

(3) $$\begin{array}{c} {}[\mathrm{Recovery}\mathrm{of}\mathrm{laccase}\mathrm{activity}(\%)=\mathrm{Total}\mathrm{activity}\mathrm{of}\mathrm{immobilized}\\ \mathrm{laccase}(U)÷\mathrm{Total}\mathrm{activity}\mathrm{of}\mathrm{free}\mathrm{laccase}(U)\times 100] \end{array}$$

(4) $$\begin{array}{c} {}[\mathrm{Specific}\mathrm{activity}(U/mg\mathrm{protein})=\mathrm{Activity}\mathrm{of}\mathrm{immobilized}\\ \mathrm{laccase}(U/g\mathrm{BC})÷\mathrm{Protein}\mathrm{loading}(mg/g\mathrm{BC}] \end{array}$$

### Reusability of the Immobilized Laccases

According to the assay protocol for laccase activity, immobilized laccases were added to initialize the first reaction. After the reaction, the immobilized enzymes were separated by filtration and then rinsed with 50 mM sodium acetate buffer (pH 5.2) until the washing water was colorless. The second reaction was started by adding the washed immobilized laccase in a new reaction medium composed of 0.4 mM ABTS and 50 mM sodium acetate buffer (pH 5.2). The repeated reactions were run several times to measure the residual activity of the immobilized laccase. Triplicate experiments for each sample were carried out and mean values are given. ANOVA was performed and *P* value is given.

### Protein Assay

Protein was determined according to Bradford’s method (Bradford, 1976) by using BSA as a standard. The amount of bound protein in BC membrane was determined indirectly by comparing the difference between the total amount of protein introduced into the reaction medium and the amount of protein in the filtrate. Very little protein could be found in the washing solutions after immobilization and it could therefore be ignored. Protein loading was calculated using equation (5). Triplicate experiments for each sample were carried out and mean values are given. ANOVA was performed and *P* value is given.

(5) $$\begin{array}{c} {}[\mathrm{Protein}\mathrm{loading}(mg/g\mathrm{BC})=[\mathrm{Total}\mathrm{protein}\mathrm{of}\mathrm{free}\mathrm{laccase}(\mathrm{mg})-Totalresidual protein\\ \mathrm{of}\mathrm{free}\mathrm{laccase}\mathrm{after}\mathrm{immobilization}(\mathrm{mg})]÷\mathrm{Total}\mathrm{mass}\mathrm{of}\mathrm{BC}\mathrm{support}(g)] \end{array}$$

### Characterization

Scanning electron microscopy (SEM, S-4800 Hitachi Ltd., Japan) was applied for morphologic observation. Prior to the inspection, the lyophilized BC sample was coated with gold. The structural features of BC were further investigated by X-ray diffraction using a D/Max-2550PC diffractometer at 40 kV and 200 mA. Angular scanning was continued 5° to 60° (2ɛ) at 1°/min. The crystalline index (CrI^XRD^) was calculated using an empirical method for native cellulose: CrI^XRD^ = (I_200_–I_am_)/I_200_ × 100%, where I_200_ is the maximum intensity of the (200) lattice diffraction and I_am_ is the intensity diffraction at 2ɛ = 18° (Focher et al., 2001). The pore size and surface area of the dry BC sponge sample were determined with a Brunauer-Emmett-Teller (BET) surface area analyzer (ASAP 2020 – Physisorption Analyzer, Micromeritics Instrument Corporation, GA). Before determination, the accurately weighed freeze-dried samples were put in sample tubes and heated at 90°C under vacuum for 3 h to remove surface moisture and other contaminants and cooled to room temperature before the BET analysis. The BET analysis was done with a relative vapor pressure of 0.05–0.25. The BET pore size and surface area were determined with N_2_ adsorption at 77 K. Quintuplicate experiments for each sample were carried out and mean values are given.

## Results and Discussion

### Structural Properties of BC Membranes

**FIGURE 1:**
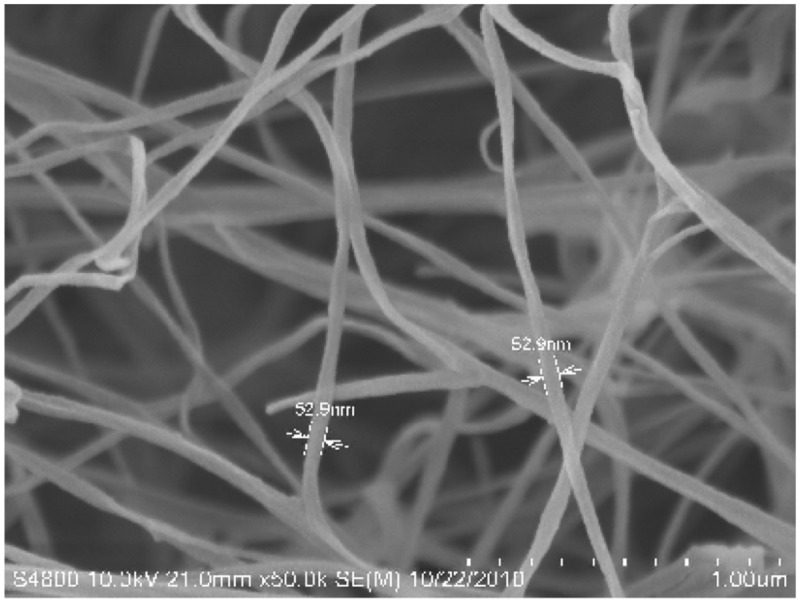
**Scanning electron micrograph of the foam-like bacterial cellulose (BC) membrane produced by *Gluconacetobacter xylinus* (×50,000)**.

**FIGURE 2:**
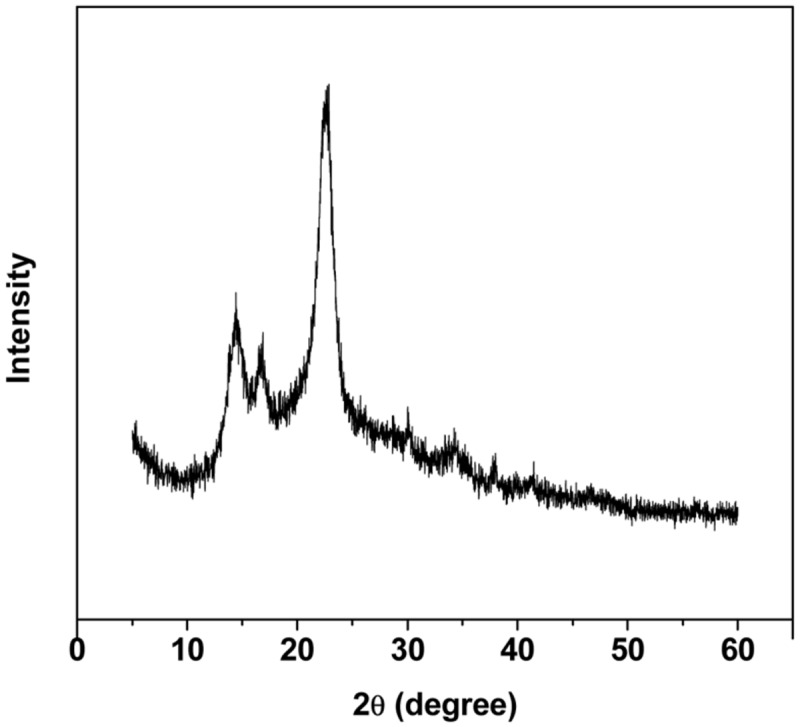
**X-ray diffraction pattern of the BC membrane**.

**Figure 1** shows the scanning electron micrograph of the spongy BC membrane produced by *G. xylinus*. From the picture, it could be seen that the BC membrane had a reticulated structure consisting of ultrafine cellulose fibrils with a diameter of less than 100 nm, which resulted in large surface area. The pore size and the surface area of the BC membranes were determined by using the BET method. The results showed that the average pore size, the pore volume and the surface area of the BC sponge were 11.5 nm, 0.25 cm^3^/g, and 72.2 m^2^/g, respectively. This structure will generate a hydrogel (a hydrated membrane) when the BC sponge is placed in water or aqueous solution of solutes. Sokolnicki et al. (2006) showed that BC would have a high swelling ratio of 17.9 and a high porosity of 0.94, indicating that defined cylindrical pores do not exist while instead micro-channels of varying sizes are present. It is concluded that the high surface area and porous features of BC membrane should provide micro-channels to entrap enzyme and improve the contact area exposed to enzyme protein molecules, and hence increase the amount of bound protein. The structural features of BC were also studied by using XRD (**Figure 2**). **Figure 2** shows three diffraction peaks at around 15.0°, 16.5°, and 22.8°, the presence of which were attributed to the typical profile of cellulose I (natural cellulose) in crystalline form (Atalla and Vanderhart, 1984). A crystallinity of 86.9% was obtained by calculation of the crystalline index (CrI^XRD^).

### Effects of Size of BC Membranes on Laccase Immobilization

Lyophilized spongy BC membranes were cut into several pieces with the sizes (mm × mm) of 7 × 7, 10 × 10, 15 × 15, and 20 × 20. An experiment was performed to study the effects of the size of BC on laccase immobilization by putting these BC pieces in 10 mL of a laccase-containing aqueous solution that was incubated statically for 24 h. Triplicate experiments for each sample were carried out and mean values are given in **Table 1**.

**Table 1:** Effects of size of bacterial cellulose (BC) membranes on laccase immobilization using only physical adsorption. Triplicate experiments for each sample were carried out and mean values are given.

BC membrane size (mm × mm)	7 × 7	10 × 10	15 × 15	20 × 20
BC mass (mg)	2.4 ± 0.1	4.4 ± 0.1	8.7 ± 0.2	18.5 ± 0.3
Adsorption ratio of enzyme (%)	13.4 ± 0.3	18.6 ± 0.5	22.1 ± 0.4	39.2 ± 0.6
Recovery of laccase activity (%)	2.3 ± 0.1	3.7 ± 0.2	4.5 ± 0.1	8.0 ± 0.3
Activity of immobilized laccase (U/g BC)	9.7 ± 0.4	8.4 ± 0.3	6.9 ± 0.2	5.9 ± 0.2
Protein loading (mg protein/g BC)	3.2 ± 0.2	3.0 ± 0.2	2.7 ± 0.1	2.1 ± 0.1
Specific activity (U/mg protein)	3.0 ± 0.3	2.8 ± 0.3	2.6 ± 0.1	2.8 ± 0.1

**Table 1** shows that the adsorption ratio of enzyme and the recovery of the laccase activity increased gradually with larger size of the membrane support, while the activity of immobilized laccase and the protein loading decreased with every gram of carrier. The highest values for adsorption ratio of enzyme (39.2%) and recovery of laccase activity (8.0%) were obtained with a BC size of 20 mm × 20 mm. The best results for the activity of immobilized laccase (9.7 U/g BC) and protein loading (3.2 mg protein/g BC) were obtained with the 7 mm × 7 mm BC membrane. In addition, the specific activity of immobilized laccase based on adsorbed protein did not show any significant difference (from 2.6 to 3.0 U/mg, *P* > 0.05) among the four sizes of BC membranes. The enzyme loading is comparable to other reported results. Arica et al. (2009) obtained an adsorption capacity of 4.9 mg/g support when using poly(GMA/EGDMA) beads as carriers. Osma et al. (2010) reported that an activity of immobilized laccase of 7.4 U/g support and a protein loading of 0.18 mg/g support were achieved when alumina (Al_2_O_3_) spherical pellets (3 mm diameter) were used as supports. This study showed that the activity of immobilized laccase, which ranged from 5.9 to 9.7 U/g BC, and the protein loading, which ranged from 2.1 to 3.2 mg protein/g BC, are comparable to those reported in the literature, indicating that the immobilization of the enzyme on BC was reasonable and satisfactory.

The reason behind the fact that the activity of the immobilized enzyme and the loaded protein decreased with increasing size of BC may be ascribed to that the amplification of enzyme protein adsorption is less than that of the BC mass when the size of the BC membranes were enlarged. Enlarging the size of BC cannot only augment the space for adsorption of enzyme molecules, but also increase the bulk and mass of BC. Through calculation, a mass amplification of 83% is obtained when the BC size was enlarged from 7 × 7 to 10 × 10 mm^2^ (BC mass increased from 2.4 to 4.4 mg), but it is only 59% for the total activity of adsorbed laccase. In a similar way, a mass amplification of 113% and an amplification of total activity of 82% are obtained when the membrane size was enlarged from 15 × 15 to 20 × 20 mm^2^ (BC mass was from 8.7 to 18.5 mg), respectively. The unbalance of amplification between adsorbed enzyme and BC mass would lead to this inconsistency.

The apparent discrepancy between adsorption ratio of enzyme and recovery of laccase activity might be ascribed to the difference in the matrices, steric hindrance of the adsorbed enzyme, differences in the methods for activity measurement, and even enzyme denaturation. Free laccase is defined as enzyme dissolved in water or buffer, leading to a quick catalytic reaction because of its easy binding with the substrate ABTS, which is also soluble in the aqueous phase during the activity assay. However, the media was changed from water for free enzyme to porous BC network filled with water for immobilized enzyme. More mass transfer resistance forms after laccase is entrapped and immobilized in the porous network of BC compared to the free enzyme. The mass transfer resistance decreases the binding efficiency between the enzyme and the substrate ABTS, and reduces the diffusion of the oxidized product of ABTS from the inside to the outside of the BC membrane. Mass transfer resistance is crucial for immobilized enzyme (Gardossi et al., 2010). Our previous research showed that the porous network of a BC hydrogel would determine the rate of entrapped drug release (Wei et al., 2011), and, vice versa, the network would control diffusion of chemicals from the outside to the inside of the BC. In this study, the obtained activity depends on the formation of product from the substrate ABTS molecule, which is required to diffuse from the outside into the network of BC to get in contact with the immobilized laccase to react and then its radical product is required to diffuse to the outside. Therefore, the activity of the immobilized laccase would depend on the diffusion coefficient and the efficiency of the substrate ABTS molecule through the highly porous network of hydrogel matrix (**Figure 1**). An analogous molecule of ABTS (548.7 Da) in terms of molecular weight (MW), Vitamin B12 (1355 Da), had a smaller diffusivity (0.72×10^-6^ cm^2^/s) in the BC membranes than in water (3.79×10^-6^ cm^2^/s) (Sokolnicki et al., 2006), indicating that ABTS and the oxidized ABTS product also should have a smaller diffusivity than in water. Transport of ABTS occurs predominately through the water in the membrane with a pore mechanism, but some molecules are retarded due to partitioning into the cellulose as well as adsorption onto the membrane, as stated in the literature (Sokolnicki et al., 2006). Therefore the smaller diffusivity of ABTS leads to the lower activity determined for immobilized enzyme than for free one, whose activity was used to calculate the adsorption ratio of enzyme. The determined lower activity of immobilized laccase as compared with free laccase would undoubtedly lead to a much less recovery of activity than the adsorption ratio of laccase, but actually the authentic activity of immobilized laccase should be higher than that obtained by using the analytic method. It could be concluded that the present analytical method for determining the activity of the BC-immobilized enzyme is not good enough since it shows much lower activity recovery compared to the actual adsorption ratio of enzyme. This situation is not like that in research on nanoparticle-immobilized laccase, whose reactive behavior with substrate is similar to that of the free enzyme.

Sokolnicki et al. (2006) studied mass transport parameters of hydrated BC membranes including solubility, diffusion and permeability coefficients, and adsorption equilibrium partition coefficients identifying the binding of solute onto the membrane scaffolding, with the goals to quantify the transport of proteins and small molecules in the cellulose membrane and to apply an understanding of the membrane structure to determine the mechanism of transport in the system. The results indicated that there exist dual transport mechanisms, for solute transport through the continuous water phase and cellulose matrix with some hindrance of molecular diffusion via fiber obstruction. With the small solute Vitamin B12, equilibrium interactions such as adsorption and solubility are also important. The permeability coefficients and the effective diffusion coefficients of the solutes decreased with increasing MW of the diffusing molecules (Sokolnicki et al., 2006). In this study, the substrate ABTS is also a small molecule solute with a MW of 548.7 Da as small as Vitamin B12. It will also have dual transport mechanisms, which determine the obtained activity of immobilized laccase. Some of the ABTS^+^ radical molecules in the catalysis may be reversibly adsorbed onto and/or absorbed into the BC membrane fibers. This entrapment or immobilization on/within the polymer, most likely at crystalline regions and solubility in the amorphous phase, influences the interfacial velocity, requiring more time to travel the length of the membrane. When all of the interactive sites are occupied and/or solubility limits reached, free diffusion endures, as stated in the literature (Sokolnicki et al., 2006).

### Effects of Amount of BC Pieces on Laccase Immobilization

Lyophilized BC membranes of 10.2 mg were cut into 1–4 pieces and were then incubated statically in 10 mL laccase-containing aqueous solution for 24 h. The experiment was performed to study the effects of the amount of BC pieces with the same gram of mass on laccase immobilization. Triplicate experiments for each sample were carried out and mean values are given in **Table 2**.

**Table 2:** Effects of amount of BC pieces of total mass of 10.2 mg on laccase immobilization using only physical adsorption. Triplicate experiments for each sample were carried out and mean values are given.

Number of BC membranes	1	2	3	4
Adsorption ratio of enzyme (%)	31.1 ± 1.5	35.7 ± 1.3	38.6 ± 1.3	42.3 ± 1.5
Recovery of laccase activity (%)	6.1 ± 0.3	6.8 ± 0.2	7.4 ± 0.3	8.6 ± 0.3
Activity of immobilized laccase (U/g BC)	5.7 ± 0.3	7.1 ± 0.3	8.5 ± 0.3	9.8 ± 0.2
Protein loading (mg protein/g BC)	2.4 ± 0.2	2.9 ± 0.1	3.2 ± 0.2	3.9 ± 0.2
Specific activity (U/mg protein)	2.3 ± 0.2	2.5 ± 0.1	2.6 ± 0.1	2.5 ± 0.1

**Table 2** indicates that four index points including adsorption ratio of enzyme (increased from 31.1 to 42.3%), recovery of laccase activity (increased from 6.1 to 8.6%), activity of immobilized laccase and protein loading all together increased gradually as the number of pieces of BC support increased. When the number of BC pieces was four, the activity of immobilized laccase and protein loading reached the highest values, i.e., 9.8 U/g BC and 3.9 mg protein/g BC, respectively. The specific activity of immobilized laccase also did not change very much among these four groups with regard to the numbers of BC pieces (from 2.3 to 2.6 U/mg). The adsorption ratio of enzyme protein ranging from 31.1 to 42.3% is in accordance with those data (protein yield ranged from 31.5 to 41.5%) reported in a literature (Rekuć et al., 2009).

The result implies that an increased number of BC pieces of the same BC mass corresponds to increased adsorption area and reaction surface based on the same mass. More enzyme protein can be easily adsorbed or entrapped by the shortened routes and more contacting surfaces of BC carriers for the diffusion of enzyme. This will also happen for ABTS diffusion during activity assay. A higher total activity obtained from the increased number of BC pieces will result in a higher activity of immobilized laccase for each gram of BC support (9.8 U/g BC was obtained by using four pieces while only 5.7 U/g BC for one piece). The reason behind the difference between the adsorption ratio of laccase and the recovery of laccase activity should be due to the diffusion distance and diffusion surface of ABTS into BC, as similar as the situation in the section of 2.2. Although agitation was used in the activity measurements, the interior environment of the BC support could not be easily disturbed by agitation because of the relatively big size of the support (still being around 4 mm × 4 mm).

### Effects of Adsorption Time on Laccase Immobilization

Freeze-dried BC membranes of 2.6 mg were incubated in 10 mL of laccase-containing aqueous solution for 0.5–8 h and 24 h. The experiment was performed to study the effects of the adsorption time on laccase immobilization. Triplicate experiments for each sample were carried out and mean values are given in **Table 3**.

**Table 3:** Effects of adsorption time on laccase immobilization using only physical adsorption. Triplicate experiments for each sample were carried out and mean values are given.

Adsorption time (h)	0.5	1	2	4	6	8	24
Adsorption ratio of enzyme (%)	27.6 ± 1.6	28.4 ± 1.4	29.9 ± 1.5	34.7 ± 1.5	34.5 ± 1.3	34.7 ± 1.5	35.1 ± 1.8
Recovery of laccase activity (%)	0.4 ± 0.1	0.7 ± 0.1	0.9 ± 0.1	1.1 ± 0.1	1.1 ± 0.1	1.2 ± 0.1	1.2 ± 0.2
Activity of immobilized laccase (U/g BC)	1.9 ± 0.1	3.0 ± 0.2	3.4 ± 0.2	4.8 ± 0.2	4.9 ± 0.3	5.1 ± 0.3	5.2 ± 0.5

**Table 3** indicates that the three index points including adsorption ratio of enzyme, recovery of laccase activity, and activity of immobilized laccase all together increased gradually with prolonged adsorption time. The adsorption ratio of enzyme and recovery of laccase activity did not change significantly after 4 h of incubation. This is probably due to that the adsorption space of the nanostructured network of the BC was nearly fully occupied after an incubation of 4 h. However, changes in the activity of immobilized enzyme only became negligible after 6 h of incubation. This result confirms that the incubation time of 24 h previously used is appropriate, which could ensure enough time to make enzyme proteins penetrate and diffuse into the internal network of BC carriers. A longer lag time than 2 h and an equilibrium time of 8 h required for laccase (64 kDa) in the membranes were observed in this study, which is consistent with that a protein with similar size as laccase, BSA (66.3 kDa), had nearly 2 h lag time and 8 h induction time (Sokolnicki et al., 2006). Solutes were found to have an induction phase followed by a free diffusion phase when the transport of BSA was evaluated in the study of permeability of BC membranes (Sokolnicki et al., 2006).

### pH Profiles of Immobilized Laccase

**FIGURE 3:**
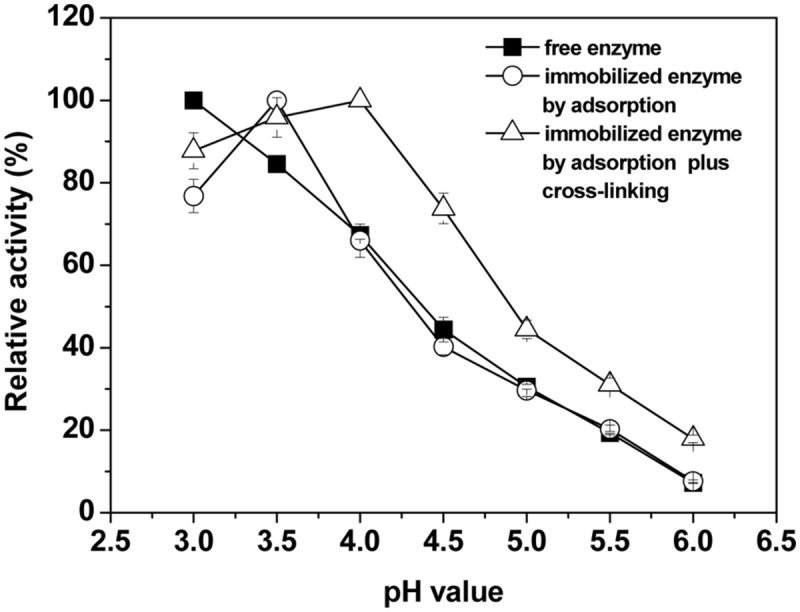
**Effects of pH on the relative activity of free and immobilized laccases.** The maximal activity was set to 100% using ABTS as substrate. Standard deviations of triplicate measurements are shown.

The effects of pH on the activity of the free and immobilized laccase were studied in the pH range from 3.0 to 6.0. **Figure 3** shows that the optimum pH value was 3.5 and 4.0 for the adsorption-immobilized and the crosslinking-immobilized laccase, respectively. For free laccase, the activity was strongly pH-dependent and had no optimum. The free laccase showed highest activity at pH 3.0 with ABTS (non-phenolic) as substrate, which is consistent with a previous study (Hong et al., 2006). However, after immobilization by physical adsorption, activity increased rapidly first then decreased as pH was increased, and the curve shape and the optimal pH were significantly changed (shifted from pH 3.0 to pH 3.5, *P* < 0.05). After immobilization by adsorption-crosslinking, the optimum value shifted to pH 4.0. In comparison with the free laccase, the crosslinking-immobilized laccase exhibited more than 90% of the maximum activity with a wider pH range between 3.0 and 4.0 (**Figure 3**). Therefore, the immobilized laccase maintained a relatively high activity over a broader pH range. This means that the laccase immobilization by adsorption-crosslinking on BC membrane could increase the pH resistance of high catalytic activity within a broader pH range, and this property is crucial in practical applications, e.g., in the treatment of textile industrial waste eﬄuent.

The pH optimum of fungal laccase is strictly dependent on the type of substrate used. For non-phenolic substrates, for example, ABTS and 2,4,5- trimethoxybenzyl alcohol, the activity of fungal laccase from *T. versicolor* always increases with decreasing pH (monotonic pH profiles), but for phenols bell-shaped pH profiles with an optimum at pH 4 are exhibited all the time (Hong et al., 2006). It would be interesting to investigate pH profiles of other substrates in the future, especially phenolic compounds, to see if the BC-immobilized enzyme will show a higher pH optimum than pH 4 as with the ABTS used in this study. It is known that the optimum pH for an immobilized enzyme shifting to a higher or lower pH depends upon surface charges of the support (Zhang et al., 2012), or in other words, depends upon the ionic interaction between enzyme and charged surface of the support (Wang et al., 2010). The shift in optimum pH toward a less acidic pH value upon immobilization may be due to the difference in the hydronium ion concentration of the micro-structures of the polymeric support and in the bulk of the solution. The BC support has many hydroxyl groups on its inner surface, which could attract more hydrogen ions from reaction solution. It could be deduced that the pH value for the immobilized enzyme experienced a lower pH in the support pores of BC than in the reaction media, and therefore shifted to higher pH values (Zhang et al., 2012).

As compared to the native free enzyme, the fact that the crosslinking-immobilized laccase showed broadening in the pH-activity profile means that the immobilization methods may preserve the enzyme activity and limit the transition of enzyme conformation in a wider pH range by the formation of multipoint non-covalent interactions, as reported in the literature (Arica et al., 2009; Majeau et al., 2010). This may be the reason why the optimum pH of immobilized enzyme was between pH 3.5 and 4.0, while free enzyme showed a monotonic pH profile.

### Temperature Profiles of Immobilized Laccase

**FIGURE 4:**
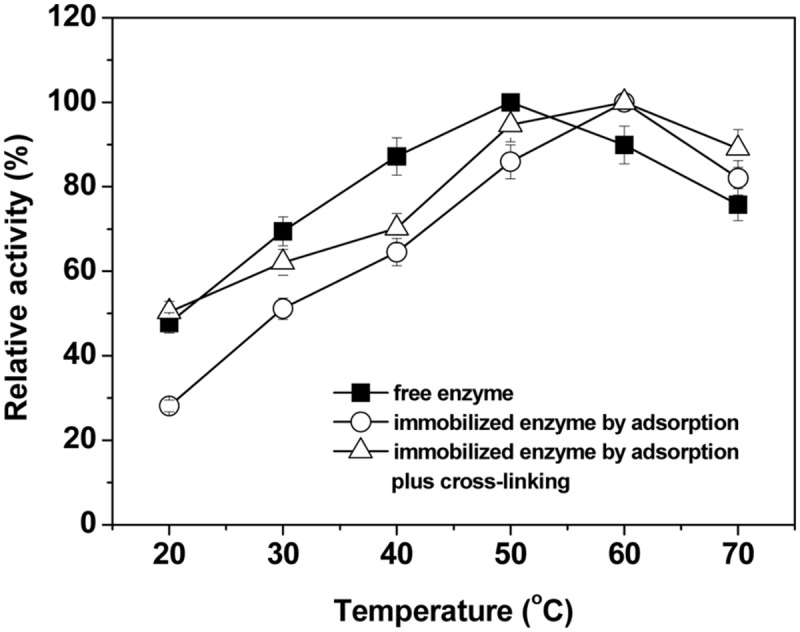
**Effects of temperature on the relative activity of free and immobilized laccase.** The maximal activity was set to 100% using ABTS as substrate. Standard deviations of triplicate measurements are shown.

The effects of temperature on the activity of the free and immobilized laccases were also studied in the temperature range of 20–70°C and data were shown in **Figure 4**. **Figure 4** shows that the activities of either free or immobilized laccase increased gradually first with increasing temperature until optima and thereafter decreased with further increases of temperature. The optimal catalytic temperature of immobilized laccases was higher than that of free form and shifted from 50°C to 60°C (**Figure 4**). The shift of the optimum temperature indicates an increase in the thermal stability of the immobilized laccases, and this should be related to the change of physical and chemical properties of the immobilized enzymes. In particular for adsorption-immobilization, the non-covalent multipoint chelate interactions between laccase and support may restrict the degrees of freedom of the molecular structure of the enzyme, thus protecting it to some extent from denaturation at high temperature (Wang et al., 2010; Bayramoglu et al., 2012). Furthermore, compared with the free and adsorption-immobilized laccase, the crosslinking-immobilized enzyme exhibited a significantly broader profile (*P* < 0.05), and the relative activity was maintained at over 90% within the temperature range of 50–70°C. The covalent bond formation after crosslinking via amino groups of the immobilized laccase and hydroxyl groups of BC might also reduce the conformational flexibility and may result in higher activation energy for the enzyme molecule to reorganize the proper conformation for the binding of the substrate (Arica et al., 2009; Zhang et al., 2012).

### Reusability of Immobilized Laccase

**FIGURE 5:**
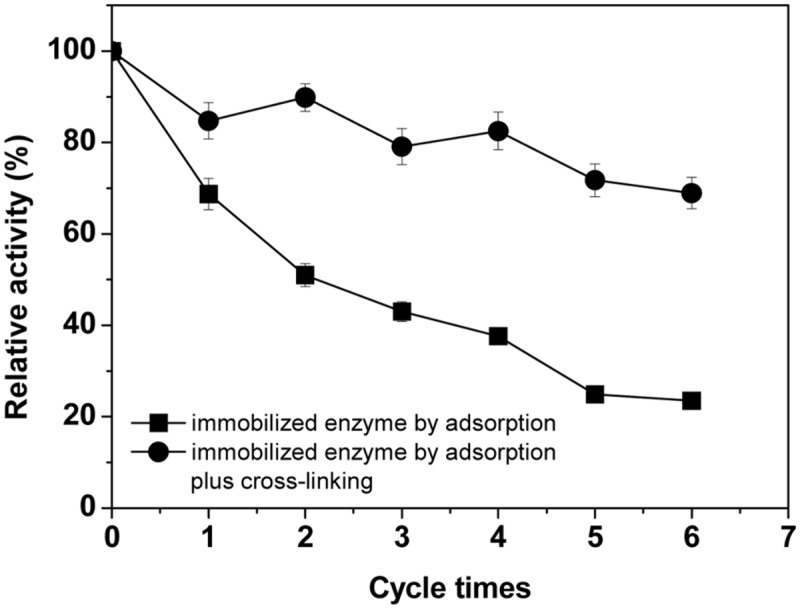
**The reuse stability of the immobilized laccases.** Standard deviations of triplicate measurements are shown.

The reusabilities of laccase immobilized on BC membrane by the two methods were studied in this test. The original activity of the immobilized enzyme was defined as 100% with a value of 6.2 U/g BC. As seen in **Figure 5**, significant improvement (*P* < 0.01) of operational stability could be observed with the crosslinking-immobilized laccase compared to that immobilized only by physical adsorption. The former enzyme retained 69% of the original activity after recycling seven times, while only 23% residual activity remained for the latter. This is due to that the adsorption method just supplied a relative poor interaction between the enzyme and the carrier, whereas the cross-linking treatment by glutaraldehyde after adsorption significantly decreased the loss of enzyme protein during reaction because of diffusion, and subsequently improved the operational stability of enzyme. This result is comparable with that in a research, which showed 78% of the initial activity of the immobilized *T. villosa* laccase remained after four cycles (Zhang et al., 2012). The results also showed that there was a gradual decrease after every cycle because of loss of a small amount of enzyme immobilized on BC or possible inactivation of enzyme in each cycle.

As Sokolnicki et al. (2006) indicated for permeability of BC membranes, the dual transport mechanism for solute transport in the continuous water phase (pore mechanism) and cellulose matrix with some hindering of molecular diffusion via fiber obstruction should also be applicable here with respect to the diffusion of laccase immobilized in BC membranes. BSA has a size of 66.3 kDa and has a smaller diffusivity (0.09×10^-6^ cm^2^/s) in the BC membranes than in water (0.59×10^-6^ cm^2^/s) (Sokolnicki et al., 2006), which implies that a laccase of around 64 kDa (Hong et al., 2002) should similarly have a small diffusivity. Diffusion with the zero solubility of BSA in the membrane, hydrodynamic and entropic exclusions account for the high degree of hindrance of BSA due to its large MW and hydrodynamic radius (Sokolnicki et al., 2006). Like BSA, no adsorption occurring within or on the fiber networks, diffusion of the laccase within BC to outside occurs entirely through the water-filled channels (pore mechanism) and thereafter would lead to the activity loss of the adsorption-immobilized enzyme. But the hindered diffusion of laccase in the BC membrane due to fiber obstruction would restrict the loss of activity of the immobilized enzyme and ensure that it was possible to reuse the enzyme at least four times and maintain around 40% of the initial activity (**Figure 5**).

## Conclusion

Bacterial cellulose and fungal laccase are biomacromolecules generated from microorganisms and in this study complete integration of these two molecules by using a simple and convenient method was performed successfully for the first time. Two different immobilization methods including physical adsorption and adsorption-crosslinking were compared. The results indicated that natural nanostructured BC membranes were possible to use as a support to immobilize *T. versicolor* laccase. The crosslinking-immobilized enzyme exhibited broader pH operation range of high catalytic activity and higher running stability compared with the free and adsorption-immobilized enzymes, which is crucial for practical applications. The immobilized enzyme proved to be stable and retained 69% of the original activity after recycling seven times. The improved reusability of laccase by immobilization would help cut down the running cost and make the enzymatic process economically feasible in an industrial scale. The advantages of using BC membranes as supports for enzyme immobilization include the following points. First, an open fiber network is ideal for immobilizing enzymes capable of reacting with diffusing species while allowing passage of soluble products with slight retardation. Second, the BC membrane has excellent wet mechanical strength compared to other polysaccharide hydrated membranes, which is a benefit for long-term running in film bioreactors. Novel applications of the BC-immobilized enzyme tentatively include active packaging, construction of biosensors for determination of phenols, as well as establishment of bioreactors for treatment of waste water/industrial eﬄuent and for other catalytic reactions. Currently, BC is not cheap, but many research groups, including our, are making important attempts to decrease the production cost of the material. It is believed that BC will become inexpensive in a not far future.

## Author Contributions

FH designed and coordinated the study and revised the manuscript. LC and MZ contributed the preparation of the manuscript. MZ carried out the experiments and analyzed the results. All authors read and approved the final manuscript.

## Conflict of Interest Statement

The authors declare that the research was conducted in the absence of any commercial or financial relationships that could be construed as a potential conflict of interest.
