# Erratum: Evaluation of Fungal Laccase Immobilized on Natural Nanostructured Bacterial Cellulose

**DOI:** 10.3389/fmicb.2015.01327

**Published:** 2015-11-16

**Authors:** Frontiers Production Office

**Affiliations:** Frontiers Production Office, FrontiersLausanne, Switzerland

**Keywords:** bacterial cellulose, laccase, adsorption, cross-linking, immobilization

Reason for Erratum:

Due to a typesetting error, the first sentence, in the section *Immobilization of Laccase* was published incorrectly. The correct sentence should read:

In general, laccase immobilization by adsorption was carried out (Hong et al., 2010) as follows: freeze-dried BC samples was placed into 10 mL of laccase aqueous solution (1000 U/L, pH 5.0) and incubated at 23°C and 80 rpm for 5 min, then was placed in a 4°C fridge statically for 24 h to equilibrate.

The publisher apologizes for this mistake, the original article was updated. This error does not change the scientific conclusions of the article in any way.
